# Succinylation-annotated genes in AMI: multi-omics and single-cell prioritization of ASGR2 and NPL

**DOI:** 10.3389/fcvm.2026.1836786

**Published:** 2026-06-25

**Authors:** Jie Yu, Xu Ma, Jing Fang, Yingying Liu, Cong Wang, Shuxia Shi, Kaile Wang, Yunlun Li, Lei Zhang

**Affiliations:** 1 First Clinical Medical College, Shandong University of Traditional Chinese Medicine, Jinan, Shandong, China; 2 Department of Hemodialysis, The Second Affiliated Hospital of Shandong University of Traditional Chinese Medicine, Jinan, Shandong, China; 3 The Third Department of Cardiovascular Disease, Affiliated Hospital of Shandong University of Traditional Chinese Medicine, Jinan, Shandong, China

**Keywords:** acute myocardial infarction, bioinformatics, biomarker, monocyte, succinylation

## Abstract

**Background:**

Current diagnostic and prognostic biomarkers for acute myocardial infarction (AMI) remain limited. Protein succinylation may provide novel biomarker candidates for AMI.

**Methods:**

Weighted gene co-expression network analysis (WGCNA) was applied to GSE66360 to identify AMI-related modules, and succinylation-annotated genes were retrieved from GeneCards. Using GSE66360 as the training set and GSE48060, GSE60993, and GSE59867 as validation sets, we evaluated 107 predefined machine-learning pipelines and assessed hub genes by differential expression and ROC analysis. Immune infiltration and gene-cell correlations were assessed with CIBERSORT. Single-cell transcriptomics examined hub-gene expression across monocyte subsets in plaque rupture (PR) and non-plaque rupture (NPR) cases, and exploratory pseudotime analysis assessed monocyte-state heterogeneity. ELISA was used to measure circulating protein levels.

**Results:**

Integrating WGCNA with GeneCards yielded 18 succinylation-annotated AMI genes. Among the evaluated pipelines, Stepglm[both] + plsRglm and Stepglm[backward] + plsRglm showed relatively favorable external validation performance. ROC and differential expression analyses prioritized ASGR2 and NPL as exploratory candidate biomarkers. Both genes correlated positively with monocytes, particularly classical monocytes. Classical monocytes were more abundant in NPR than PR samples. ASGR2 and NPL showed exploratory expression trends along an inferred pseudotime axis. ELISA showed elevated plasma levels of ASGR2 and NPL in AMI patients compared with control individuals.

**Conclusion:**

ASGR2 and NPL were identified as hypothesis-generating candidate biomarkers associated with acute myocardial infarction. Given the limited training sample size and the high number of evaluated machine-learning pipelines, these findings remain exploratory and require independent prospective validation before clinical translation. Their enrichment in monocytes, particularly classical monocytes, suggests a potential association with monocyte-related inflammatory remodeling.

## 1 Background

Despite advances in diagnosis and therapy, acute myocardial infarction (AMI) remains a leading cause of death worldwide. Timely and accurate diagnosis is essential for effective treatment and prognostication. At present, electrocardiography (ECG) and myocardial biomarkers are the principal diagnostic tools, but both have limitations (1,2). ECG changes can be nonspecific, particularly in NSTEMI; over one-third of patients may show only T-wave inversion or ST-segment depression (3). High-sensitivity cardiac troponin (hs-cTn) improves sensitivity, yet elevations also occur in diverse ischemic and non-ischemic settings (4). In addition, the absence of universally accepted hs-cTn decision thresholds contributes to variable sensitivity and specificity, increasing the risk of misclassification (5). Thus, more sensitive and specific biomarkers are needed to improve early diagnosis and clinical management.

Succinylation is a recently characterized lysine post-translational modification in which succinyl-CoA adds a four-carbon, negatively charged group to the lysine amine (6). This modification influences diverse biological processes (7) and has been implicated in AMI pathogenesis. For example, inhibiting succinate dehydrogenase alleviates ischemia-reperfusion injury in the context of SIRT5 depletion (8), and succinyl-CoA synthetase knockout reduces inflammation and slows heart-failure progression. Moreover, suppressing succinylation can reduce endothelial apoptosis (9). These observations suggest that succinylation-annotated genes may serve as biomarkers and therapeutic targets in AMI. However, the specific genes involved and their mechanistic contributions remain incompletely defined.

**Figure 1:**
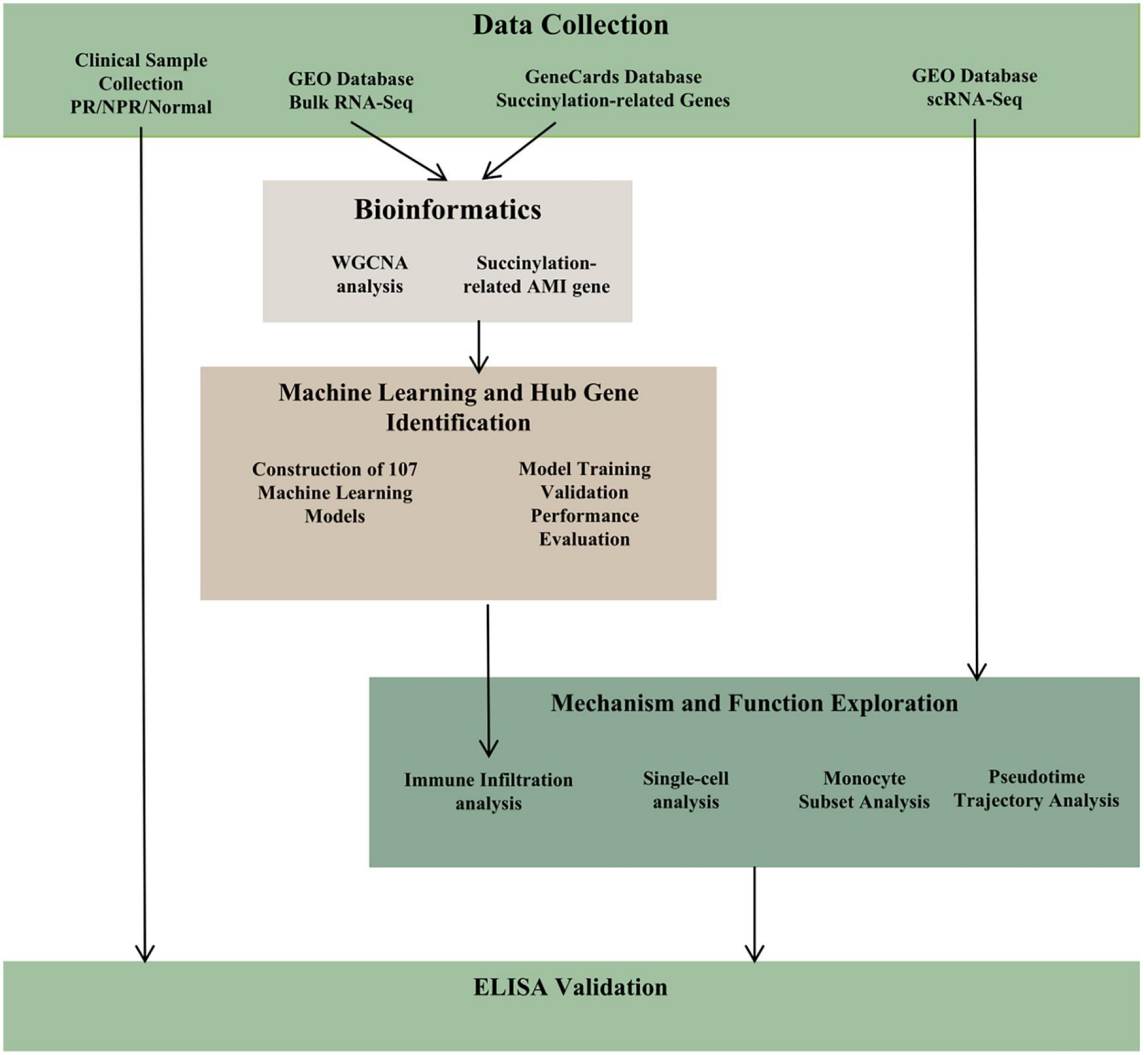
Workflow diagram.

To investigate succinylation-linked regulation in AMI, we analyzed patient RNA-seq data from GEO and succinylation-annotated genes from GeneCards. WGCNA was used to identify AMI modules enriched for succinylation-annotated genes, followed by external validation using 107 machine-learning models and ROC analysis. Given the central role of monocytes in AMI-associated inflammation, we examined associations between candidate genes and monocyte subsets, including differences between plaque-rupture and non-plaque-rupture AMI. Pseudotime analysis assessed expression dynamics during monocyte development, and ELISA validated key targets. Figure 1 Summarizes the study workflow.

## 2 Materials and methods

### 2.1 Data collection and preprocessing

Succinylation-annotated genes were obtained from GeneCards (https://www.genecards.org/) using the keyword “succinylation modification” (10). Bulk transcriptomic datasets (GSE66360, GSE48060, GSE60993, GSE59867) and scRNA-seq data (GSE269269) were downloaded from GEO (https://www.ncbi.nlm.nih.gov/geo/) (11). Sample characteristics are summarized in Table 1. GSE66360 served as the training set (12), while GSE48060, GSE60993, and GSE59867 were used for validation (13–15). GSE269269 was analyzed for single-cell profiling (16).

**Table 1:** Dataset information.

ID	platform	Sample type	Sample size (n), total (case:control)	Data type
GSE66360	GPL570	Blood	99 (49:50)	Microarray
GSE48060	GPL570	Blood	52 (31:21)	Microarray
GSE60993	GPL6884	Blood	24 (7:17)	Microarray
GSE59867	GPL6244	Blood	157 (111:46)	Microarray
GSE269269	GPL24676	Blood	10 (5:5)	scRNA-seq

Platform-specific annotation files were used to map probes to genes, and expression data were normalized with the ScaleData function. The GSE269269 cohort includes 5 plaque-rupture (PR) and 5 non-plaque-rupture (NPR) AMI samples. scRNA-seq preprocessing in Seurat included conversion to sparse matrices, quality control (nCount_RNA > 500; nFeature_RNA 1,000–4,000; mitochondrial reads <20%; erythrocyte contamination <1%), normalization (NormalizeData), variable feature selection (FindVariableFeatures), and scaling (ScaleData). Dimensionality reduction used PCA, and cluster annotation relied on Cell Marker 2.0 (17).

### 2.2 Clinical sample collection

Following the 2023 ESC Guidelines for acute coronary syndromes, 20 patients with AMI were enrolled based on standard diagnostic criteria. Coronary angiography was used to classify patients into plaque-rupture (PR) and non-plaque-rupture (NPR) groups. Ten control participants with no clinically significant abnormalities on physical examination served as controls. All participants provided written informed consent in accordance with the Declaration of Helsinki. The study was approved by the Ethics Committee of the Affiliated Hospital of Shandong University of Traditional Chinese Medicine on June 29, 2023 (Approval No. (2023) Lun Shen (296)-KY).

### 2.3 Weighted gene Co-expression network analysis (WGCNA)

WGCNA (18) was performed on GSE66360 using the WGCNA R package to identify gene modules associated with AMI. Genes with standard deviation >0.5 were retained, and goodSamplesGenes was used to remove low-quality samples/genes. Candidate soft-thresholding powers were evaluated using the pickSoftThreshold function in the WGCNA package. The soft-thresholding power was selected by jointly considering the scale-free topology fit index and mean network connectivity. Specifically, we chose the lowest power at which the scale-free topology fit index exceeded 0.8 while the network retained acceptable mean connectivity, in accordance with the standard WGCNA recommendation to balance scale-free approximation and network sparsity. The adjacency matrix was transformed into a topological overlap matrix (TOM) to quantify pairwise gene connectivity. Modules were detected using dynamic tree cutting (minimum module size = 50), and similar modules were merged with mergeCloseModules. Module-trait relationships were assessed as Pearson correlations between module eigengenes and the binary clinical trait (AMI = 1, normal = 0), with corresponding *P* values calculated according to the standard WGCNA procedure. The module-trait relationship heatmap was used to identify modules most relevant to AMI.

### 2.4 Functional enrichment analysis

Succinylation-annotated AMI genes were annotated by KEGG (19–21) and Gene Ontology (GO) enrichment (BP, CC, MF) using clusterProfiler (22). Terms with *p* < 0.01 were considered significant and visualized.

### 2.5 Hub gene identification

To identify hub genes with potential diagnostic value, we constructed a predefined machine-learning framework based on 18 succinylation-annotated candidate genes associated with acute myocardial infarction. Eleven commonly used machine-learning algorithms were included: Lasso, Ridge, Elastic Net, stepwise logistic regression, support vector machine, linear discriminant analysis, glmBoost, plsRglm, random forest, XGBoost, and Naive Bayes.

The 107 candidate models represented predefined single-step and two-step machine-learning pipelines rather than a simple arithmetic combination of algorithms. In single-step pipelines, one algorithm was directly applied to the 18 candidate genes for classification model fitting. In two-step pipelines, the algorithm before the “+” sign was used for feature selection or dimensionality reduction, whereas the algorithm after the “+” sign was used for downstream classifier construction. For example, Stepglm[both] + plsRglm indicates that bidirectional stepwise generalized linear modeling was first used for feature selection, followed by construction of a plsRglm classifier using the selected variables. The complete list of the 107 candidate model pipelines is provided in Supplementary Table S1.

All feature selection, model training, hyperparameter determination, and prediction threshold determination were performed exclusively in the training dataset GSE66360. The external validation datasets were used only for independent performance evaluation and were not involved in feature selection, model fitting, hyperparameter optimization, or threshold determination, thereby reducing the risk of information leakage.

For Lasso, Ridge, and Elastic Net, the regularization parameter *λ* was selected using 10-fold cross-validation within the training dataset using cv.glmnet. The final model was fitted using *λ*.min, defined as the value of *λ* that produced the minimum mean cross-validated error. For Lasso and Elastic Net-based feature selection, features with nonzero coefficients at *λ*.min were retained for subsequent model construction. For Elastic Net, the mixing parameter *α* was prespecified according to the model setting.

For XGBoost, 5-fold cross-validation was used within the training dataset to determine the optimal number of boosting rounds. In each fold, the boosting round with the minimum test log-loss was identified, and the final number of rounds was selected as the most frequently occurring optimal round across the five folds. The remaining XGBoost parameters were fixed as max.depth = 2 and eta = 1.

For glmBoost, internal 10-fold cross-validation was used to estimate the optimal boosting iteration number, and the final model was refitted using the selected mstop, with a lower bound of 40 iterations. Features with nonzero model coefficients were retained. Linear discriminant analysis was trained using the cross-validation procedure implemented in the caret package. For plsRglm, cross-validation was performed with nt = 10, and sparse PLS logistic regression was used; features with nonzero coefficients were retained. Random forest was fitted with ntree = 1,000 and nodesize = 5, with variable importance, proximity, and forest estimation enabled. Random forest-based feature selection was performed using the var.select function, and the variables returned as topvars were retained. Stepwise logistic regression performed feature selection based on the Akaike information criterion, and variables retained in the final model were used for downstream modeling. Support vector machine and Naive Bayes did not apply additional embedded feature-selection thresholds and were trained using the input feature set provided to the algorithm.

Differential expression of the eight hub genes between groups was assessed using the Wilcoxon test, followed by Benjamini-Hochberg false discovery rate correction for multiple comparisons. Adjusted *P* values <0.05 were considered statistically significant.

### 2.6 Immune cell correlation analysis

CIBERSORT was used to estimate the fractions of 22 immune cell subtypes in GSE66360 (23). Comparisons of immune cell proportions between groups were performed using the Wilcoxon rank-sum test, followed by Benjamini-Hochberg correction for multiple comparisons. Spearman correlation analysis was conducted to evaluate the relationships between hub gene expression and immune cell infiltration levels. Multiple testing correction for correlation analyses was performed using the Benjamini–Hochberg procedure. Results were visualized using ggplot2.

### 2.7 Single-cell transcriptomics

Cells were clustered with UMAP and annotated using Cell Marker 2.0. Hub-gene expression was visualized with FeaturePlot, and stacked bar plots compared monocyte subset proportions between PR and NPR samples.

### 2.8 Exploratory pseudotime analysis

Monocytes from PR and NPR samples were subsetted. Differentially expressed genes were identified using dispersionTable (mean_expression ≥ 0.05; dispersion_empirical≥0.5). Pseudotime ordering was inferred using Monocle (v2.34.0). Trajectory-dependent genes (*q*-value <0.01) were visualized by heatmaps.

### 2.9 Enzyme-linked immunosorbent assay (ELISA)

Fasting peripheral blood samples were collected into EDTA tubes and centrifuged within 30 min at 1,000 × g for 15 min at 4 °C. Plasma was collected and stored at −20 °C until analysis. Plasma concentrations of ASGR2 and NPL were measured using commercially available ELISA kits according to the manufacturers' instructions. The kit information, including manufacturer, catalog number, sensitivity, and detection range, is provided in Table 2. All samples were measured under the same experimental conditions.

**Table 2:** Characteristics of ELISA kits used in this study.

Parameter	ASGR2	NPL
Manufacturer	ELK Biotechnology	ELK Biotechnology
Catalog number	ELK3154	ELK0324
Analytical sensitivity	26 pg/mL	0.097 ng/mL
Detection range	62.5–4,000 pg/mL	0.32–20 ng/mL
Intra-assay CV	<8%	<8%
Inter-assay CV	<10%	<10%
Linearity	79%–92%	95%–104%
Recovery	95%–107%	80%–95%

## 3 Results

### 3.1 Identification of succinylation-annotated core gene modules and functional enrichment analysis

**Figure 2:**
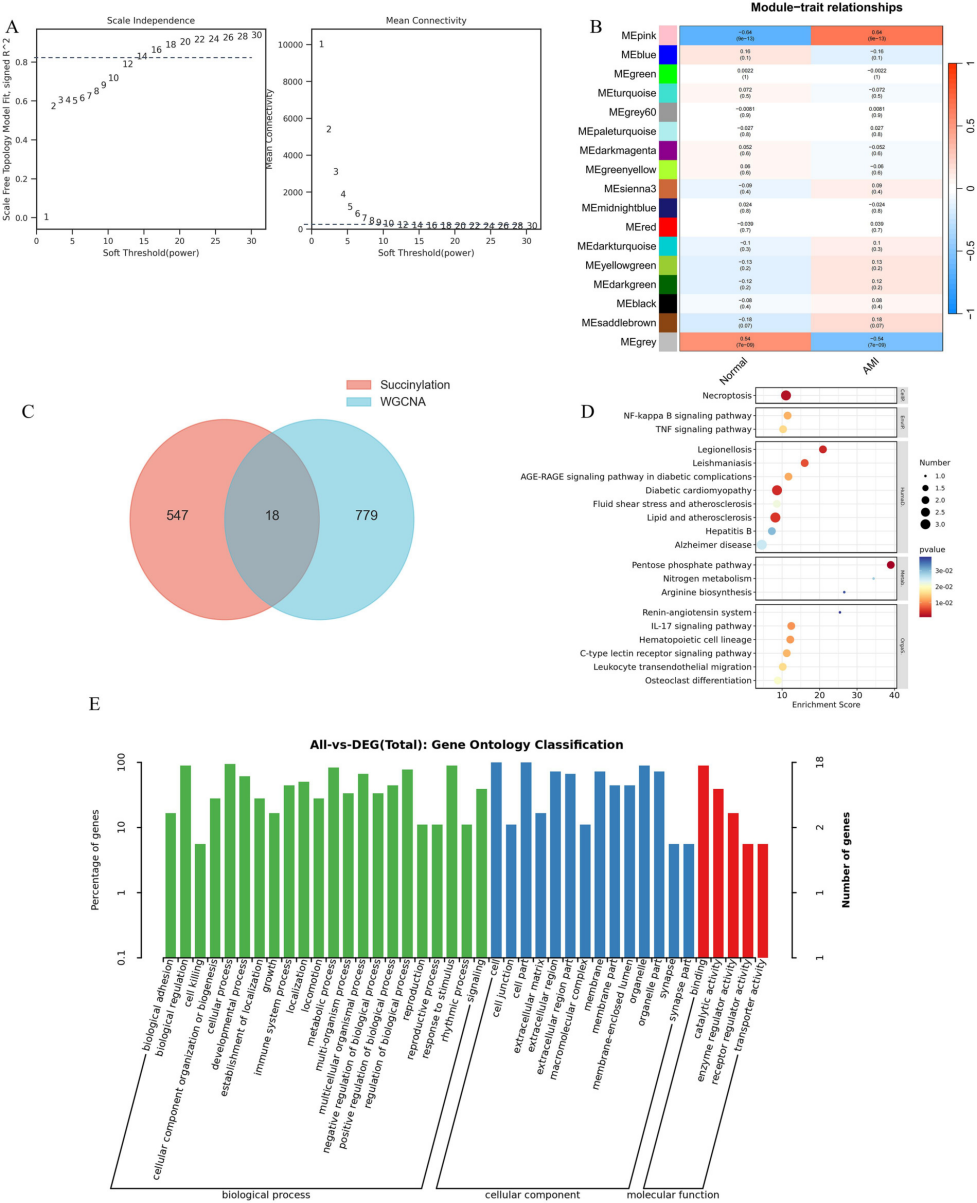
Identification of key modules and functional enrichment analysis in AMI. **(A)** Selection of the soft-thresholding power for WGCNA based on both the scale-free topology fit index and mean connectivity. *β* = 14 was selected because it was the lowest power at which the scale-free topology fit index exceeded 0.8 while maintaining acceptable network connectivity. **(B)** Module-trait relationship analysis identified the pink module as most significantly correlated with AMI. **(C)** Venn diagram intersection of succinylation-annotated genes (*n* = 565) with AMI-associated genes (*n* = 797) yielded 18 candidate genes for further analysis. **(D,E)** Functional enrichment of succinylation-annotated AMI genes revealed.

WGCNA of GSE66360 identified 17 modules (Figure 2A) using a soft-threshold power of *β* = 14. The pink module showed the strongest association with AMI (*r* = 0.64, *P* = 9 × 10^−13^) and contained 797 genes (Figure 2B). From GeneCards, 565 succinylation-annotated genes were retrieved; intersecting these with the pink module yielded 18 succinylation-annotated AMI genes (Figure 2C). KEGG (Figure 2D) and GO (Figure 2E) enrichment analyses of these 18 genes identified six KEGG pathways (e.g., pentose phosphate pathway, necroptosis, lipid metabolism and atherosclerosis; *P* < 0.01). GO analysis indicated significant enrichment in 10 biological processes (e.g., pentose-phosphate shunt, cellular response to UV-A), 10 cellular components (e.g., tertiary granule lumen, extracellular space), and 10 molecular functions (e.g., protease binding, endopeptidase activity) (all *P* < 0.01). These results suggest that succinylation-annotated genes may contribute to metabolic reprogramming, inflammatory responses, and plaque destabilization during AMI progression.

### 3.2 Identification of hub genes using multi-model machine learning

Based on the 18 succinylation-annotated AMI candidate genes, we constructed and evaluated 107 predefined machine learning pipelines. These pipelines included single-step classification models and two-step models composed of a feature selection algorithm followed by a downstream classifier. All variable selection, model fitting, and parameter determination were performed in the GSE66360 training cohort, whereas the external validation cohorts were used only for independent performance evaluation.

**Figure 3:**
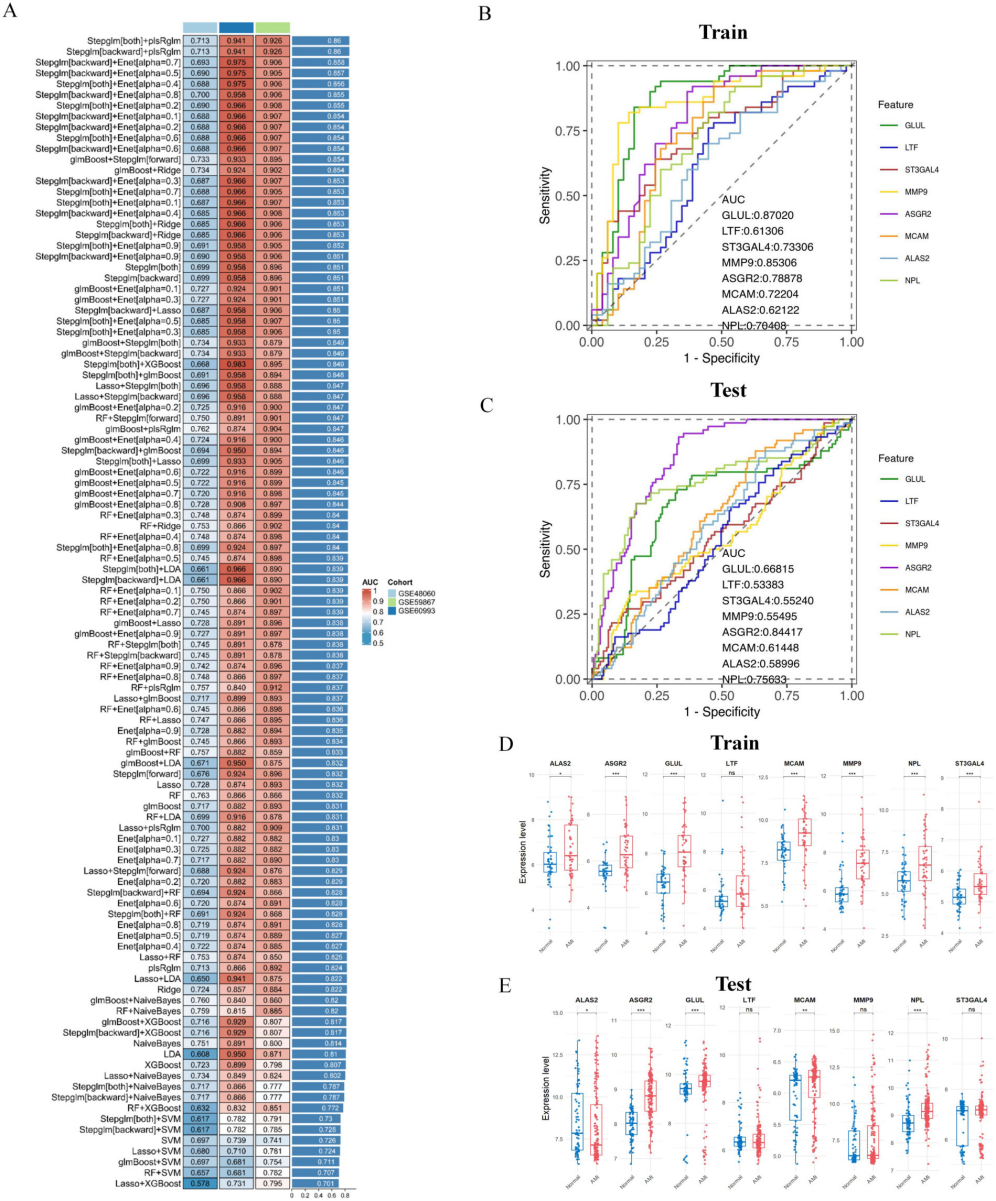
Machine learning-based identification and validation of hub genes in AMI. **(A)** Performance evaluation of 107 algorithm combinations derived from 11 machine learning models. The Stepglm[both] + plsRglm and Stepglm[backward] + plsRglm models achieved the highest mean AUC values, establishing them as optimal diagnostic classifiers. **(B,C)** ROC curve analysis of the 8 candidate hub genes in **(B)** the training cohort and **(C)** the independent validation cohort. ASGR2 and NPL demonstrated superior diagnostic performance in both cohorts. **(D,E)** Violin plots illustrating the expression distribution of the 8 hub genes in **(D)** AMI patients vs. **(E)** healthy controls. ASGR2 and NPL showed the most significant differential expression.

Among the 107 candidate machine learning pipelines, Stepglm[both] + plsRglm and Stepglm[backward] + plsRglm showed favorable performance across the three independent external validation cohorts, with mean external validation AUC values of 0.86 (Figure 3A). The AUC values for each individual external validation cohort are provided in Supplementary Table S2. The two models jointly identified eight hub genes, including GLUL, LTF, ST3GAL4, MMP9, ASGR2, MCAM, ALAS2, and NPL. We then performed single-gene ROC analysis and differential expression analysis (Figures 3B,C).

In the training dataset, ASGR2 showed an AUC of 0.789, with a sensitivity of 0.900, specificity of 0.633, and optimal cutoff value of 5.939. NPL showed an AUC of 0.704, with a sensitivity of 0.820, specificity of 0.551, and optimal cutoff value of 6.539. In the validation dataset, ASGR2 maintained good diagnostic performance, with an AUC of 0.844, sensitivity of 0.932, specificity of 0.667, and optimal cutoff value of 8.538. NPL also showed acceptable diagnostic efficacy, with an AUC of 0.756, sensitivity of 0.676, specificity of 0.830, and optimal cutoff value of 8.824. ASGR2 and NPL showed relatively stable diagnostic performance in the training and validation cohorts and were significantly differentially expressed between AMI patients and controls (Figures 3D,E); therefore, they were selected as key candidate diagnostic genes for subsequent analyses.

### 3.3 Immune infiltration and correlation analysis

**Figure 4:**
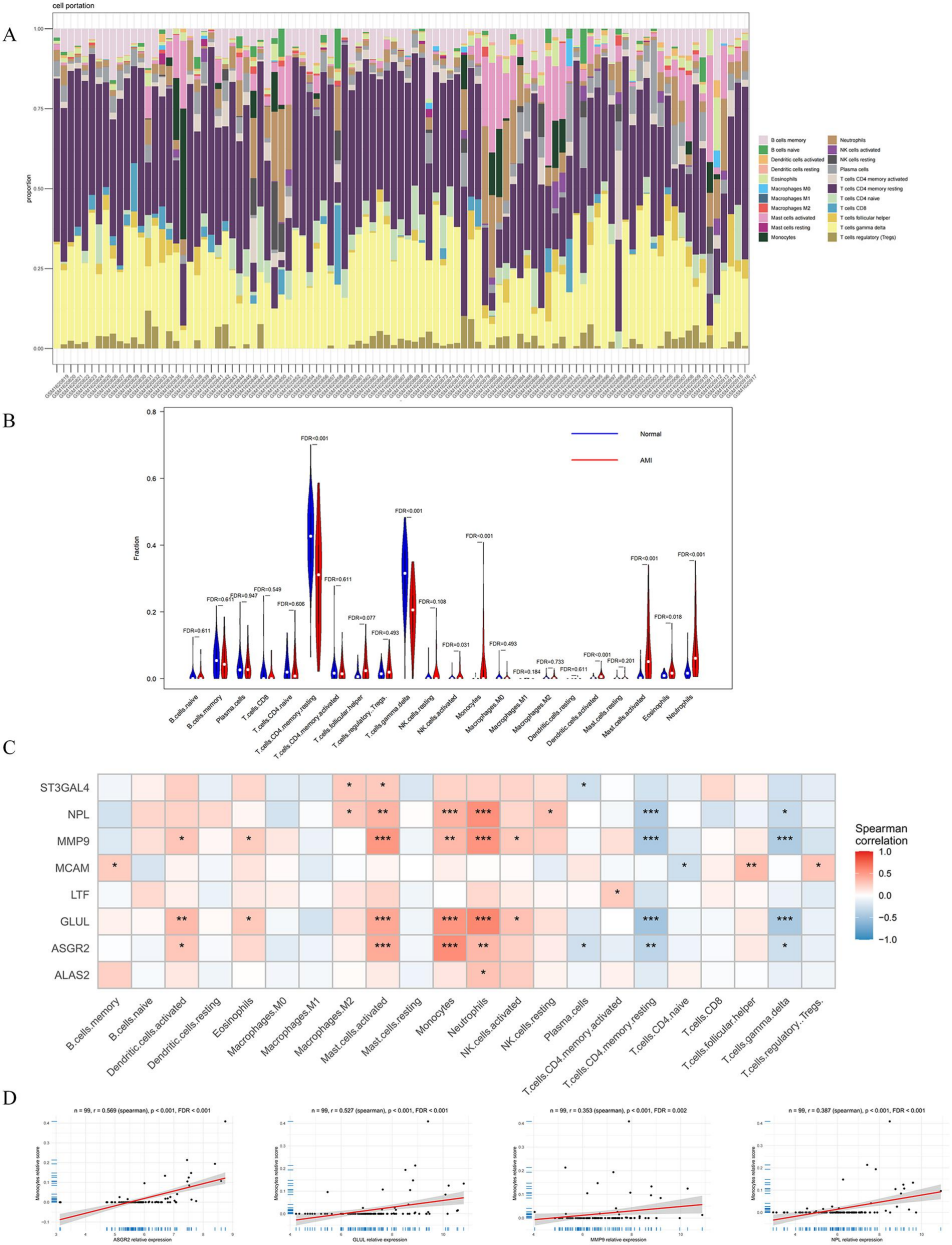
Immune infiltration landscape and hub gene correlations in AMI. **(A)** Heatmap displaying the relative abundance of 22 immune cell types across all samples. **(B)** Comparative analysis revealed significantly elevated infiltration levels of monocytes and resting dendritic cells in AMI patients vs. controls. **(C)** Correlation matrix illustrating associations between the 8 hub genes and 22 immune cell types. **(D)** Focused analysis identified three hub genes (ASGR2, GLUL, NPL) showing particularly strong correlations with monocyte infiltration (|*r*|>0.3, FDR < 0.001).

CIBERSORT was used to estimate immune cell proportions in GSE66360 (Figure 4A). Compared with controls, AMI patients showed significantly increased proportions of monocytes, resting dendritic cells, and other immune cell types after Benjamini-Hochberg correction (FDR < 0.01) (Figure 4B). Spearman correlation analysis between the 8 hub genes and 22 immune cell types showed strong positive correlations of ASGR2, GLUL, and NPL with monocytes (Figures 4C,D). These findings suggest that these hub genes may influence monocyte activity and contribute to AMI progression.

### 3.4 The single-cell-level validation of hub gene expression

**Figure 5:**
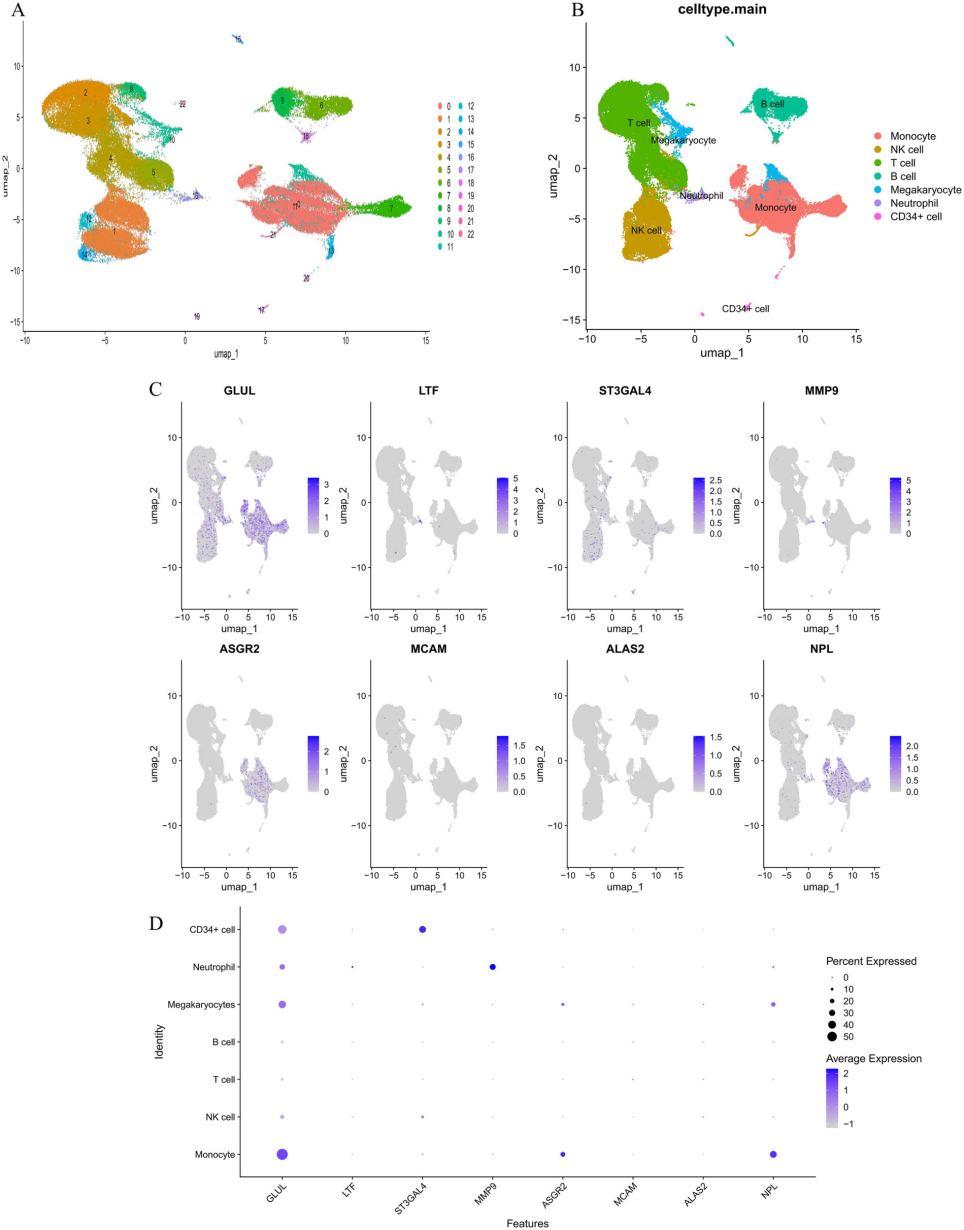
Single-cell localization of hub genes in AMI. **(A)** Unsupervised clustering of all cells identified 22 distinct clusters (umap visualization). **(B)** Annotation of the 22 clusters revealed 7 major cell types, including monocytes, NK cells, and T cells. **(C)** Illustrating the cell-type-specific distribution of hub genes, with ASGR2 and NPL predominantly localized to monocytes. **(D)** Quantifying hub gene expression levels across the 7 cell types. ASGR2 and NPL exhibited higher expression in monocytes compared to other cell types.

UMAP clustering separated cells into 22 clusters (Figure 5A). Using the CellMarker database and canonical marker gene expression patterns, the clusters were annotated as seven cell types, including monocytes, NK cells, T cells, B cells, megakaryocytes, neutrophils, and CD34+ cells (Figure 5B), and the annotation results were visualized in Supplementary Figure 1. Hub-gene expression was visualized with FeaturePlot (Figure 5C). NPL, ASGR2, and GLUL were highly expressed in monocytes, supporting the immune-infiltration results. ST3GAL4 was enriched in CD34^+^ cells, while MMP9 was mainly localized in neutrophils (Figure 5D).

### 3.5 Hub gene signatures in monocyte subsets

**Figure 6:**
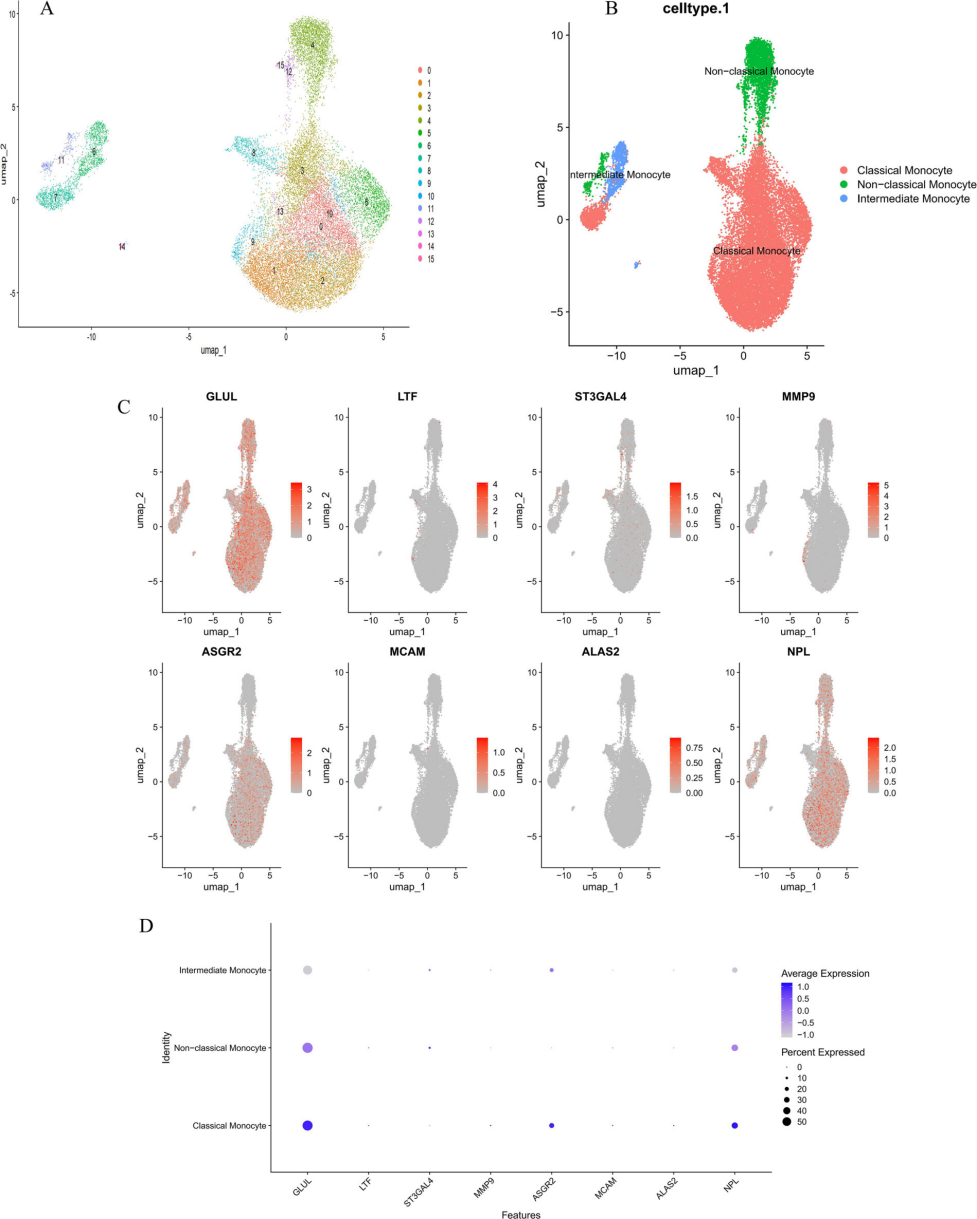
Single-cell resolution of hub gene localization in monocyte subsets. **(A)** Unsupervised clustering of monocytes identified 15 transcriptionally distinct subpopulations. **(B)** Annotation using canonical markers (CD14, FCGR3A) classified monocytes into three functional subsets. **(C)** Hub gene expression mapping revealed ASGR2 was predominantly localized to classical monocytes, while NPL showed pan-monocyte expression. **(D)** Visualization of the proportion of hub gene expression in monocytes.

Previous studies indicate that monocytes play important roles in AMI and may be influenced by succinylation. To explore this, monocytes were extracted and stratified into three subsets based on CD14 and FCGR3A (CD16a) expression (24): classical (CD14^+^CD16^—^), non-classical (CD14^—^CD16^+)^, and intermediate (CD14^+^CD16^+)^ monocytes (Figures 6A,B). Hub-gene expression differed across subsets (Figure 6C). NPL was expressed in all three subsets, with the highest levels in classical monocytes. ASGR2 was high in classical monocytes but low/absent in non-classical monocytes (Figure 6D).

### 3.6 Functional association of hub genes with plaque rupture

**Figure 7:**
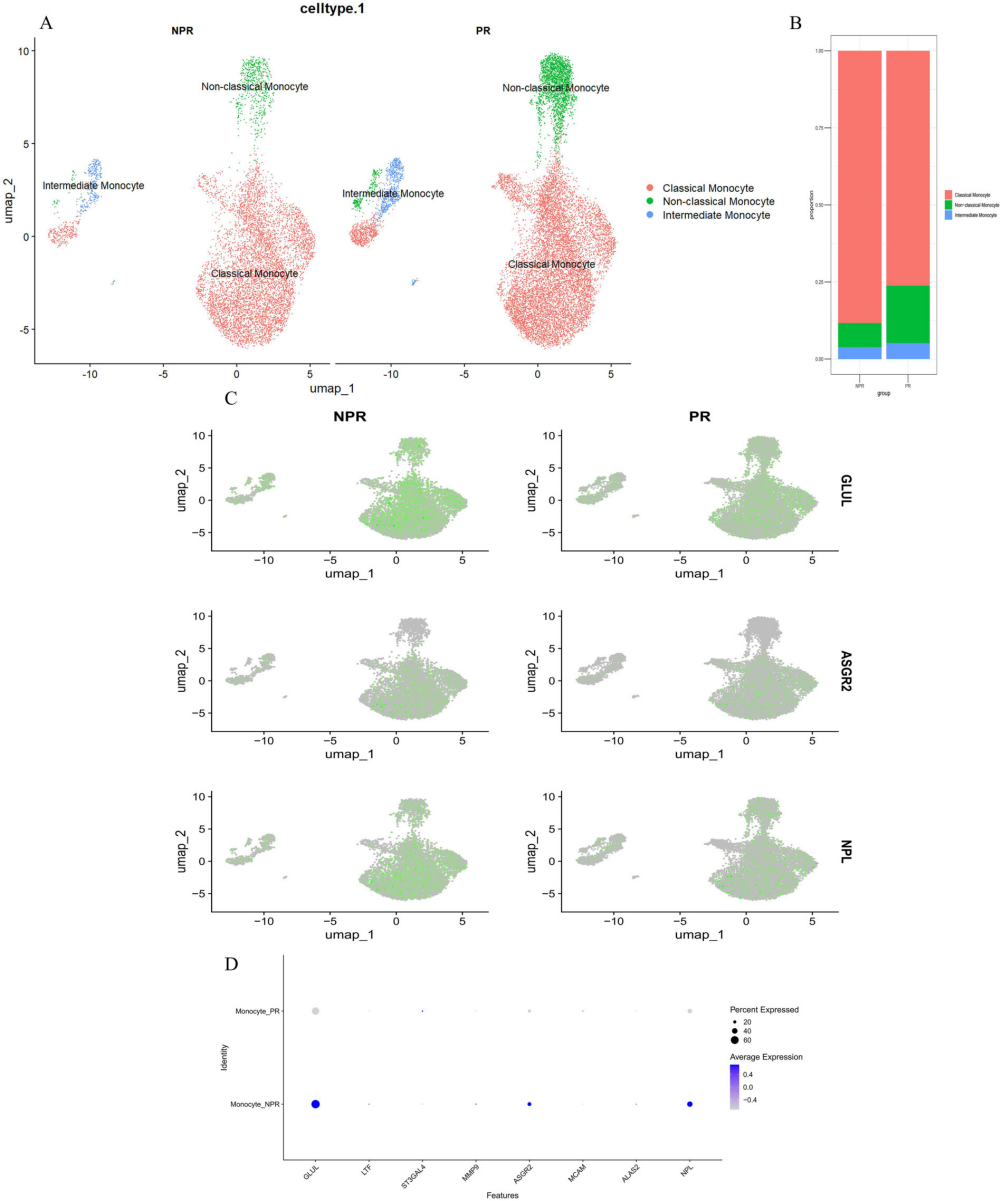
Hub gene expression dynamics in PR and NPR. **(A)** Comparing the expression profiles of Classical, Intermediate, and Non-classical monocytes between PR and NPR cohorts. **(B)** The ratio of the three monocytes is shown by histogram. **(C)** Subset-specific hub gene localization. **(D)** Visualization of the ratio of PR and NPR hub gene expression.

To explore hub-gene functions in plaque rupture (PR), monocytes were divided into PR and non-plaque rupture (NPR) groups (Figure 7A). NPR-AMI samples showed a higher proportion of classical monocytes, whereas PR samples displayed a marked increase in non-classical monocytes (Figure 7B), suggesting that shifts in monocyte subsets contribute to plaque rupture. Hub-gene expression differed between groups (Figure 7C). Notably, ASGR2 and NPL were enriched in the NPR group but reduced in the PR group (FDR < 0.01) (Figure 7D).

### 3.7 Exploratory pseudotime analysis of monocyte-state heterogeneity

Pseudotime analysis was performed as an exploratory approach to investigate monocyte-state heterogeneity in plaque rupture and non-plaque rupture samples. Given the cross-sectional nature and limited sample size of the single-cell dataset, with 5 plaque rupture and 5 non-plaque rupture samples, the inferred pseudotime results were interpreted as hypothesis-generating rather than definitive evidence of monocyte developmental trajectories or plaque rupture-specific transitional states.

**Figure 8:**
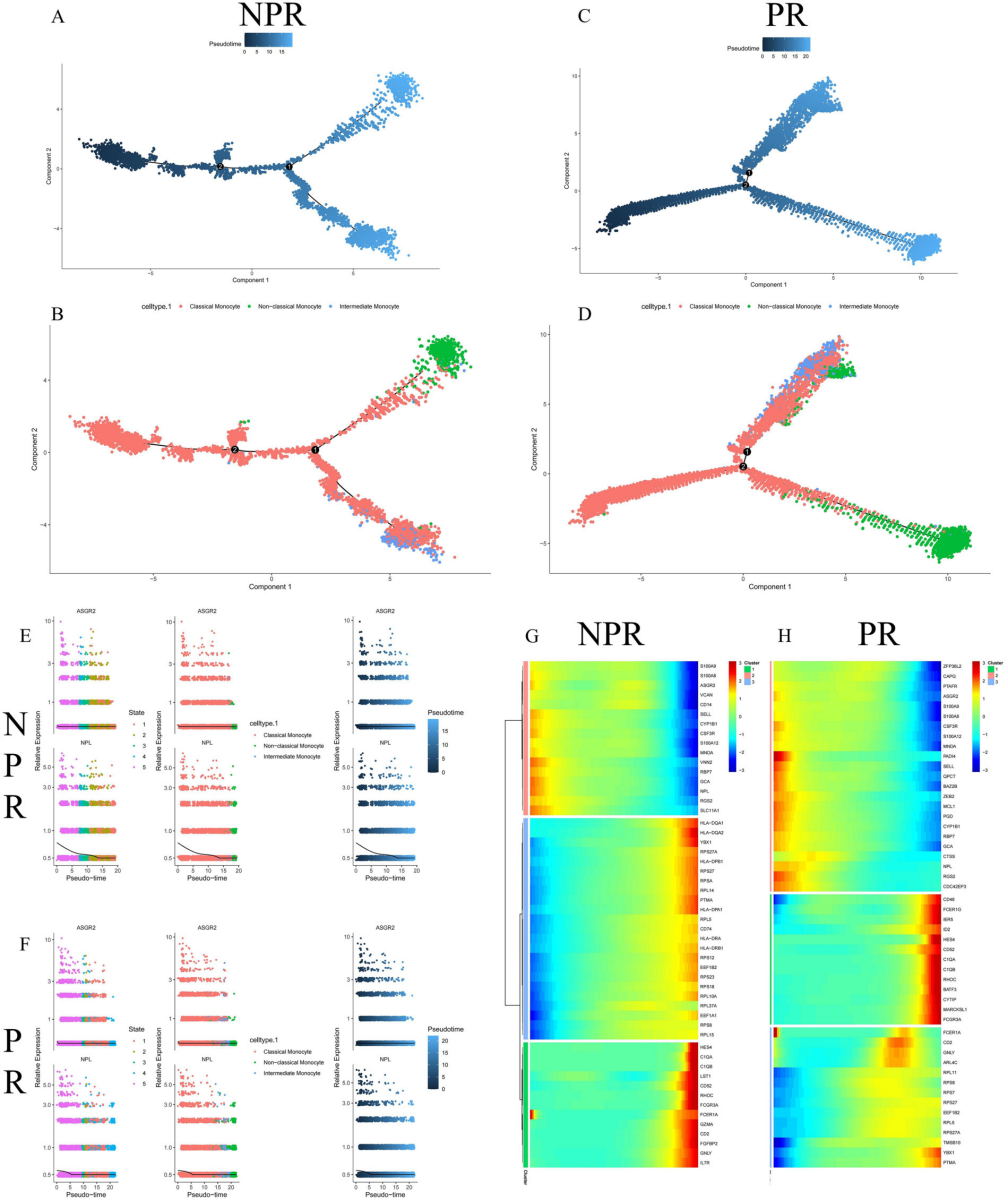
**(A–D)** Exploratory pseudotime analysis of monocytes suggested a continuum of transcriptional states spanning classical, intermediate, and non-classical monocytes. Plaque rupture and non-plaque rupture samples showed different distributions of monocyte states along the inferred pseudotime axis. **(E,F)** Dynamic expression patterns of NPL and ASGR2 along the inferred pseudotime axis. **(G,H)** Heatmaps of pseudotime-associated genes, illustrating genes with dynamic changes across the inferred monocyte-state continuum, including NPL and ASGR2.

Classical monocytes were selected as the reference starting population for pseudotime ordering based on previous human monocyte kinetic studies supporting a maturation continuum from classical to intermediate and non-classical monocytes(25,26). The inferred pseudotime axis suggested a transcriptional-state continuum involving classical, intermediate, and non-classical monocytes (Figures 8A,B).

When plaque rupture and non-plaque rupture samples were examined separately, apparent differences in monocyte-state distribution along the inferred pseudotime axis were observed (Figures 8C,D). NPL and ASGR2 expression tended to decline along the inferred pseudotime axis (Figures 8E,F), and both genes showed significant pseudotime-associated expression changes (Figures 8G,H). These findings suggest that ASGR2 and NPL may be associated with monocyte-state heterogeneity in acute myocardial infarction.

### 3.8 Measurement of plasma hub-gene protein levels

**Figure 9:**
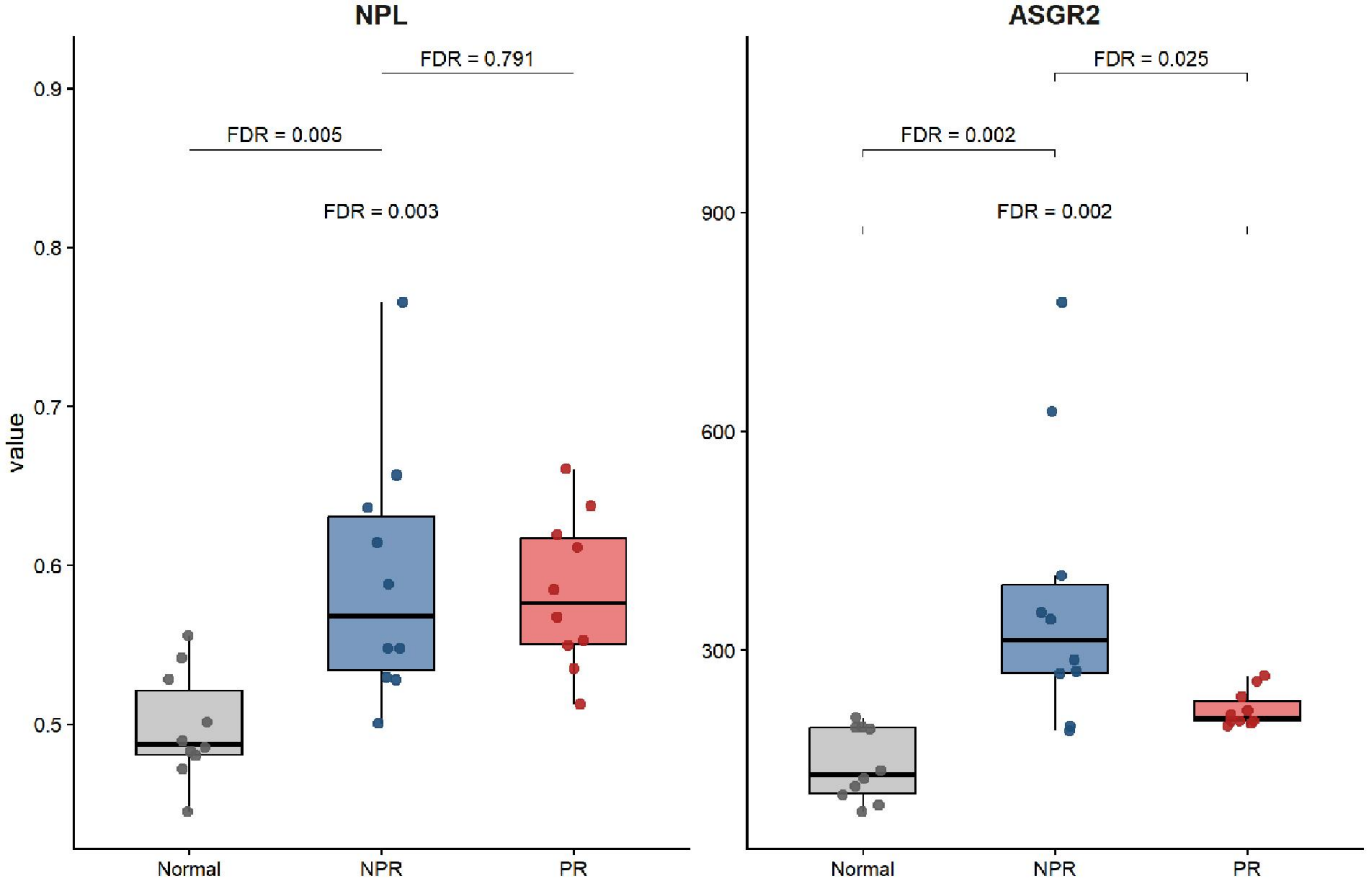
ELISA results. Comparison of ELISA levels of NPL and ASGR2 among the Normal, NPR, and PR groups. Pairwise comparisons were performed using two-sided unpaired Wilcoxon rank-sum tests, followed by Benjamini-Hochberg correction for multiple comparisons across all six pairwise tests. FDR-adjusted *P* values are shown.

To further validate hub-gene expression in plasma, NPL and ASGR2 levels were measured in control individuals (Normal), PR patients, and NPR patients. The baseline characteristics of the study population are shown in Table 3. NPL levels were significantly higher in both PR and NPR patients than in control individuals, whereas no significant difference was observed between PR and NPR groups. ASGR2 levels were also elevated in both AMI subgroups compared with control individuals. In the exploratory subgroup comparison, ASGR2 showed a nominally significant difference between NPR and PR patients (Figure 9). However, due to the limited sample size, the observed statistical significance may be prone to Type I error; therefore, this result should be regarded as exploratory and requires validation in larger cohorts.

**Table 3:** Baseline characteristics of the study population.

Variables	PR group (*n* = 10)	NPR group (*n* = 10)	Normal group (*n* = 10)	*P* value
Age, years	60.6 ± 16.3	60.5 ± 12.4	54.0 ± 13.5	0.497
Male, *n* (%)	6 (60.0)	6 (60.0)	5 (50.0)	0.873
Hyperlipidemia, *n* (%)	8 (80.0)	7 (70.0)	3 (30.0)	0.054
Diabetes mellitus, *n* (%)	5 (50.0)	5 (50.0)	2 (20.0)	0.287
Hypertension, *n* (%)	8 (80.0)	7 (70.0)	4 (40.0)	0.155
Smoking, *n* (%)	6 (60.0)	3 (30.0)	3 (30.0)	0.287
Alcohol consumption, *n* (%)	6 (60.0)	4 (40.0)	4 (40.0)	0.585
Statin use, *n* (%)	8 (80.0)	7 (70.0)	4 (40.0)	0.155
ACEI/ARB use, *n* (%)	8 (80.0)	7 (70.0)	4 (40.0)	0.155
Beta-blocker use, *n* (%)	9 (90.0)	8 (80.0)	6 (60.0)	0.271
CCB use, *n* (%)	7 (70.0)	5 (50.0)	5 (50.0)	0.581
Aspirin use, *n* (%)	8 (80.0)	6 (60.0)	4 (40.0)	0.189
P2Y12 inhibitor use, *n* (%)	3 (30.0)	2 (20.0)	0 (0.0)	0.189
Time from symptom onset to blood draw, h	0.95 ± 0.35	1.08 ± 0.58	NA	0.554

Data are presented as mean ± SD or *n* (%). ACEI/ARB, angiotensin-converting enzyme inhibitor/angiotensin receptor blocker; CCB, calcium channel blocker; NA, not applicable. Medication use refers to drugs administered or taken before blood sampling, including statins, ACEI/ARB, *β*-blockers, CCB, aspirin, and P2Y12 inhibitors. The *P* value for time from symptom onset to blood draw was calculated between the PR and NPR groups only because this variable was not applicable to the normal control group.

## 4 Discussion

Diagnosis of acute myocardial infarction (AMI) still relies primarily on ECG changes and biomarkers of myocardial injury. Although these indicators are essential for confirming AMI, their diagnostic performance may be limited in the early stage of disease, in patients with atypical symptoms, or in cases with non-diagnostic ECG findings(27). Succinylation has emerged as an important post-translational modification in cardiovascular disease, primarily affecting lysine residues and modulating core metabolic pathways (mitochondrial, amino-acid, and fatty-acid metabolism). These changes can influence healing, plaque rupture, and lesion formation (6). Most existing work focuses on SIRT family proteins (28,29), leaving other succinylation-regulated genes underexplored in AMI. To address this gap, we integrated AMI transcriptomic data with succinylation-annotated gene information and developed a computational framework comprising 107 combinations of 11 machine-learning algorithms, validated by ROC analysis.

Immune-infiltration and single-cell RNA-seq analyses showed that ASGR2 and NPL are enriched in monocytes, especially classical monocytes, and vary markedly across monocyte subsets. These genes were associated with unstable plaque rupture and linked to metabolic pathways such as the pentose phosphate and lipid pathways. ELISA analysis showed elevated plasma levels of ASGR2 and NPL in patients with AMI, supporting their potential value as candidate diagnostic biomarkers for AMI.

Monocytes are central to AMI pathogenesis. Early in plaque formation, endothelial dysfunction permits LDL-C entry into the vascular wall, recruiting monocytes via adhesion molecules (30). Monocytes differentiate into macrophages (31), ingest oxidized LDL-C, and form lipid-laden foam cells. As disease progresses, monocytes and macrophages secrete pro-inflammatory cytokines and MMPs, destabilizing plaques and increasing rupture risk (32). In our study, monocyte abundance was elevated in AMI and correlated with ASGR2 and NPL levels. We categorized monocytes into classical, intermediate, and non-classical subsets using established surface markers. Classical monocytes (≈90% of circulating monocytes) are key inflammatory effector cells that differentiate into macrophages (33). Non-classical monocytes have less defined roles in coronary artery disease but may support endothelial homeostasis (34). In the present single-cell dataset, non-plaque rupture samples showed a higher proportion of classical monocytes. Conversely, non-classical monocytes were more abundant in ruptured-plaque AMI, suggesting a potential role in post-AMI repair.

The exploratory pseudotime analysis was intended to provide a transcriptomic framework for understanding monocyte-state heterogeneity in AMI rather than to define a validated developmental trajectory. Within this framework, the dynamic changes in ASGR2 and NPL suggest that these genes may be associated with monocyte-state remodeling in AMI. Nevertheless, whether they participate directly in monocyte differentiation, activation, or plaque-related immune responses requires confirmation in larger cohorts, longitudinal sampling designs, and functional experiments.

ASGR2, a subunit of the asialoglycoprotein receptor, mediates endocytosis of glycoproteins with terminal galactose or N-acetylgalactosamine (35). ASGR2 was strongly expressed in AMI, and both training and validation cohorts showed consistent diagnostic performance (AUC > 0.7, *p* < 0.001). Its preferential expression in classical monocytes distinguishes it from non-classical monocytes and enhances its utility as a subset marker. ASGR1, another receptor subunit, can inhibit cholesterol clearance and promote coronary disease progression (36). Although ASGR2 has been linked to gastric cancer and hepatitis (37–39), its role in AMI remains largely unknown (40), warranting mechanistic studies.

NPL (N-acetylneuraminate pyruvate lyase) maintains sialic-acid homeostasis by catalyzing reversible cleavage of sialic acid into N-acetylmannosamine and pyruvate (41). Sialic acid is crucial for cell adhesion and migration (42). Dysregulation is implicated in GNE myopathy and spondyloepimetaphyseal dysplasia (43,44). NPL-knockout zebrafish exhibit severe myopathy and cardiac edema (45), and NPL-deficient mice show impaired mitochondrial function and delayed muscle regeneration (46), highlighting its role in striated muscle integrity. Our finding of elevated NPL in AMI (*p* < 0.01) aligns with a potential role as a succinylation-regulated mediator of myocardial injury.

## 5 Conclusion

Using multidimensional bioinformatics analyses, we prioritized ASGR2 and NPL as hypothesis-generating candidate biomarkers in AMI from a GeneCards-derived succinylation-annotated gene set. Both genes were associated with monocyte-related immune features, particularly classical monocytes, suggesting their potential involvement in monocyte-associated inflammatory remodeling in AMI. However, these findings should be interpreted as exploratory biomarker-prioritization results rather than clinically validated diagnostic biomarkers. Given the limited training sample size and the evaluation of 107 predefined machine-learning pipelines, the relatively high model-to-sample ratio may increase the risk of model-selection optimism and affect the robustness and generalizability of the selected candidates. Because the public datasets used in this study lacked matched clinical measurements of established biomarkers such as high-sensitivity troponin, the clinical substitutive or incremental value of ASGR2 and NPL could not be directly assessed. In addition, the link between these genes and succinylation remains annotation-based and has not been directly validated biochemically. The small ELISA validation cohort and the limited sample size of the single-cell dataset GSE269269 may further affect the robustness of our findings. Therefore, larger independent prospective multicenter cohorts, together with mechanistic and biochemical experiments, are required to validate the diagnostic utility, clinical relevance, and biological significance of ASGR2 and NPL in AMI before any clinical translation.

## Data Availability

The original contributions presented in the study are included in the article/Supplementary Material, further inquiries can be directed to the corresponding author.
