# Supplementary material for: Succinylation-annotated genes in AMI: multi-omics and single-cell prioritization of ASGR2 and NPL
Source: Front Cardiovasc Med. 2026 Jun 25;13:1836786. doi: 10.3389/fcvm.2026.1836786 (PMC13346246; doi:10.3389/fcvm.2026.1836786)
Supplement: Supplementary file 1 [file Datasheet1.docx]

***Supplementary materials***

**Supplementary table S1.** Complete list of the 107 candidate machine learning pipelines.

| Model |
| --- |
| Lasso + Stepglm [both] |
| SVM |
| glmBoost + SVM |
| Ridge |
| Lasso + SVM |
| glmBoost + Ridge |
| Enet [alpha=0.1] |
| glmBoost + Enet [alpha=0.1] |
| Enet [alpha=0.2] |
| Enet [alpha=0.3] |
| glmBoost + Enet [alpha=0.3] |
| glmBoost + Enet [alpha=0.2] |
| Enet [alpha=0.4] |
| glmBoost + Enet [alpha=0.4] |
| Lasso + glmBoost |
| Enet [alpha=0.5] |
| glmBoost |
| glmBoost + Enet [alpha=0.5] |
| Enet [alpha=0.6] |
| glmBoost + Enet [alpha=0.6] |
| glmBoost + Enet [alpha=0.7] |
| glmBoost + Enet [alpha=0.8] |
| Enet [alpha=0.8] |
| Enet [alpha=0.9] |
| Lasso |
| Enet [alpha=0.7] |
| glmBoost + Enet [alpha=0.9] |
| glmBoost + Lasso |
| Lasso + plsRglm |
| glmBoost + plsRglm |
| glmBoost + Stepglm [forward] |
| Lasso + Stepglm [forward] |
| RF + SVM |
| Stepglm [forward] |
| plsRglm |
| RF + Ridge |
| RF + Enet [alpha=0.1] |
| RF + plsRglm |
| RF + Stepglm [forward] |
| RF + Enet [alpha=0.2] |
| RF + Enet [alpha=0.3] |
| RF + Enet [alpha=0.6] |
| RF + Lasso |
| RF + Enet [alpha=0.7] |
| RF + Enet [alpha=0.5] |
| RF + glmBoost |
| RF + Enet [alpha=0.9] |
| RF + Enet [alpha=0.4] |
| RF + Enet [alpha=0.8] |
| RF + Stepglm [both] |
| RF + Stepglm [backward] |
| Stepglm [both] + Ridge |
| Stepglm [backward] + Ridge |
| Stepglm [both] + plsRglm |
| Stepglm [backward] + plsRglm |
| Stepglm [both] + Enet [alpha=0.9] |
| Stepglm [backward] + Enet [alpha=0.9] |
| Stepglm [both] + Enet [alpha=0.1] |
| Stepglm [backward] + Enet [alpha=0.1] |
| Stepglm [both] + Enet [alpha=0.8] |
| Stepglm [backward] + Enet [alpha=0.8] |
| Stepglm [both] + Enet [alpha=0.2] |
| Stepglm [backward] + Enet [alpha=0.2] |
| Stepglm [both] + Lasso |
| Stepglm [backward] + Lasso |
| Stepglm [both] + Enet [alpha=0.6] |
| Stepglm [backward] + Enet [alpha=0.6] |
| Stepglm [both] + Enet [alpha=0.7] |
| Stepglm [backward] + Enet [alpha=0.7] |
| Lasso + Stepglm [backward] |
| Stepglm [both] |
| Stepglm [backward] |
| glmBoost + Stepglm [both] |
| glmBoost + Stepglm [backward] |
| Stepglm [both] + Enet [alpha=0.4] |
| Stepglm [backward] + Enet [alpha=0.4] |
| Stepglm [both] + Enet [alpha=0.3] |
| Stepglm [backward] + Enet [alpha=0.3] |
| Stepglm [both] + glmBoost |
| Stepglm [backward] + glmBoost |
| Stepglm [both] + Enet [alpha=0.5] |
| Stepglm [backward] + Enet [alpha=0.5] |
| glmBoost + RF |
| RF |
| Stepglm [both] + SVM |
| Stepglm [backward] + SVM |
| Lasso + RF |
| Stepglm [both] + RF |
| LDA |
| glmBoost + LDA |
| RF+LDA |
| Stepglm [both]+LDA |
| Stepglm [backward]+LDA |
| Lasso + LDA |
| Stepglm [backward] + RF |
| XGBoost |
| Lasso+XGBoost |
| glmBoost+XGBoost |
| RF+XGBoost |
| Stepglm[both]+XGBoost |
| Stepglm[backward]+XGBoost |
| NaiveBayes |
| Lasso+NaiveBayes |
| glmBoost+NaiveBayes |
| RF+NaiveBayes |
| Stepglm[both]+NaiveBayes |
| Stepglm[backward]+NaiveBayes |

**Supplementary table S2.** AUC performance of top-performing machine learning models in the three external validation cohorts.

| Model | GSE48060  AUC | GSE60993  AUC | GSE59867  AUC |
| --- | --- | --- | --- |
| Lasso+Stepglm[both] | 0.695852534562212 | 0.957983193277311 | 0.887974931453192 |
| SVM | 0.696620583717358 | 0.739495798319328 | 0.740795142969056 |
| glmBoost+SVM | 0.696620583717358 | 0.680672268907563 | 0.75430865648257 |
| Ridge | 0.723502304147465 | 0.857142857142857 | 0.884449667058363 |
| Lasso+SVM | 0.680491551459293 | 0.710084033613445 | 0.780552291421857 |
| glmBoost+Ridge | 0.734254992319508 | 0.92436974789916 | 0.901880141010576 |
| Enet[alpha=0.1] | 0.726574500768049 | 0.882352941176471 | 0.881903642773208 |
| glmBoost+Enet[alpha=0.1] | 0.726574500768049 | 0.92436974789916 | 0.901096748922836 |
| Enet[alpha=0.2] | 0.720430107526882 | 0.882352941176471 | 0.882882882882883 |
| Enet[alpha=0.3] | 0.725038402457757 | 0.882352941176471 | 0.882491186839013 |
| glmBoost+Enet[alpha=0.3] | 0.726574500768049 | 0.92436974789916 | 0.900705052878966 |
| glmBoost+Enet[alpha=0.2] | 0.725038402457757 | 0.915966386554622 | 0.900117508813161 |
| Enet[alpha=0.4] | 0.721966205837174 | 0.873949579831933 | 0.884645515080298 |
| glmBoost+Enet[alpha=0.4] | 0.723502304147465 | 0.915966386554622 | 0.899529964747356 |
| Lasso+glmBoost | 0.717357910906298 | 0.899159663865546 | 0.893262828045437 |
| Enet[alpha=0.5] | 0.71889400921659 | 0.873949579831933 | 0.888562475518997 |
| glmBoost | 0.717357910906298 | 0.882352941176471 | 0.892871132001567 |
| glmBoost+Enet[alpha=0.5] | 0.721966205837174 | 0.915966386554622 | 0.898550724637681 |
| Enet[alpha=0.6] | 0.720430107526882 | 0.873949579831933 | 0.890520955738347 |
| glmBoost+Enet[alpha=0.6] | 0.721966205837174 | 0.915966386554622 | 0.899138268703486 |
| glmBoost+Enet[alpha=0.7] | 0.720430107526882 | 0.915966386554622 | 0.898159028593811 |
| glmBoost+Enet[alpha=0.8] | 0.728110599078341 | 0.907563025210084 | 0.896592244418331 |
| Enet[alpha=0.8] | 0.71889400921659 | 0.873949579831933 | 0.890716803760282 |
| Enet[alpha=0.9] | 0.728110599078341 | 0.882352941176471 | 0.894437916177047 |
| Lasso | 0.728110599078341 | 0.873949579831933 | 0.893458676067372 |
| Enet[alpha=0.7] | 0.717357910906298 | 0.882352941176471 | 0.889933411672542 |
| glmBoost+Enet[alpha=0.9] | 0.726574500768049 | 0.890756302521008 | 0.897179788484136 |
| glmBoost+Lasso | 0.728110599078341 | 0.890756302521008 | 0.896004700352526 |
| Lasso+plsRglm | 0.700460829493088 | 0.882352941176471 | 0.90912651782217 |
| glmBoost+plsRglm | 0.761904761904762 | 0.873949579831933 | 0.90442616529573 |
| glmBoost+Stepglm[forward] | 0.732718894009217 | 0.932773109243697 | 0.895025460242852 |
| Lasso+Stepglm[forward] | 0.688172043010753 | 0.92436974789916 | 0.875832354093224 |
| RF+SVM | 0.65668202764977 | 0.680672268907563 | 0.782412847630239 |
| Stepglm[forward] | 0.675883256528418 | 0.92436974789916 | 0.895808852330591 |
| plsRglm | 0.712749615975422 | 0.865546218487395 | 0.892479435957697 |
| RF+Ridge | 0.752688172043011 | 0.865546218487395 | 0.902271837054446 |
| RF+Enet[alpha=0.1] | 0.749615975422427 | 0.865546218487395 | 0.901684292988641 |
| RF+plsRglm | 0.757296466973886 | 0.840336134453782 | 0.912455934195065 |
| RF+Stepglm[forward] | 0.749615975422427 | 0.890756302521008 | 0.900705052878966 |
| RF+Enet[alpha=0.2] | 0.749615975422427 | 0.865546218487395 | 0.900900900900901 |
| RF+Enet[alpha=0.3] | 0.748079877112135 | 0.873949579831933 | 0.898942420681551 |
| RF+Enet[alpha=0.6] | 0.745007680491551 | 0.865546218487395 | 0.898354876615746 |
| RF+Lasso | 0.746543778801843 | 0.865546218487395 | 0.895417156286721 |
| RF+Enet[alpha=0.7] | 0.745007680491551 | 0.873949579831933 | 0.896788092440266 |
| RF+Enet[alpha=0.5] | 0.745007680491551 | 0.873949579831933 | 0.898354876615746 |
| RF+glmBoost | 0.745007680491551 | 0.865546218487395 | 0.892675283979632 |
| RF+Enet[alpha=0.9] | 0.741935483870968 | 0.873949579831933 | 0.896004700352526 |
| RF+Enet[alpha=0.4] | 0.748079877112135 | 0.873949579831933 | 0.898354876615746 |
| RF+Enet[alpha=0.8] | 0.748079877112135 | 0.865546218487395 | 0.896788092440266 |
| RF+Stepglm[both] | 0.745007680491551 | 0.890756302521008 | 0.877790834312573 |
| RF+Stepglm[backward] | 0.745007680491551 | 0.890756302521008 | 0.877790834312573 |
| Stepglm[both]+Ridge | 0.685099846390169 | 0.966386554621849 | 0.90638464551508 |
| Stepglm[backward]+Ridge | 0.685099846390169 | 0.966386554621849 | 0.90638464551508 |
| Stepglm[both]+plsRglm | 0.712749615975422 | 0.941176470588235 | 0.925577751664708 |
| Stepglm[backward]+plsRglm | 0.712749615975422 | 0.941176470588235 | 0.925577751664708 |
| Stepglm[both]+Enet[alpha=0.9] | 0.691244239631336 | 0.957983193277311 | 0.905405405405405 |
| Stepglm[backward]+Enet[alpha=0.9] | 0.689708141321045 | 0.957983193277311 | 0.90638464551508 |
| Stepglm[both]+Enet[alpha=0.1] | 0.686635944700461 | 0.966386554621849 | 0.906580493537015 |
| Stepglm[backward]+Enet[alpha=0.1] | 0.688172043010753 | 0.966386554621849 | 0.906972189580885 |
| Stepglm[both]+Enet[alpha=0.8] | 0.698924731182796 | 0.92436974789916 | 0.896592244418331 |
| Stepglm[backward]+Enet[alpha=0.8] | 0.700460829493088 | 0.957983193277311 | 0.90599294947121 |
| Stepglm[both]+Enet[alpha=0.2] | 0.689708141321045 | 0.966386554621849 | 0.90755973364669 |
| Stepglm[backward]+Enet[alpha=0.2] | 0.688172043010753 | 0.966386554621849 | 0.90677634155895 |
| Stepglm[both]+Lasso | 0.698924731182796 | 0.932773109243697 | 0.905405405405405 |
| Stepglm[backward]+Lasso | 0.686635944700461 | 0.957983193277311 | 0.905797101449275 |
| Stepglm[both]+Enet[alpha=0.6] | 0.688172043010753 | 0.966386554621849 | 0.906580493537015 |
| Stepglm[backward]+Enet[alpha=0.6] | 0.688172043010753 | 0.966386554621849 | 0.906580493537015 |
| Stepglm[both]+Enet[alpha=0.7] | 0.688172043010753 | 0.966386554621849 | 0.905405405405405 |
| Stepglm[backward]+Enet[alpha=0.7] | 0.692780337941628 | 0.974789915966387 | 0.90560125342734 |
| Lasso+Stepglm[backward] | 0.695852534562212 | 0.957983193277311 | 0.887974931453192 |
| Stepglm[both] | 0.698924731182796 | 0.957983193277311 | 0.896200548374461 |
| Stepglm[backward] | 0.698924731182796 | 0.957983193277311 | 0.896200548374461 |
| glmBoost+Stepglm[both] | 0.734254992319508 | 0.932773109243697 | 0.879357618488053 |
| glmBoost+Stepglm[backward] | 0.734254992319508 | 0.932773109243697 | 0.879357618488053 |
| Stepglm[both]+Enet[alpha=0.4] | 0.688172043010753 | 0.974789915966387 | 0.905797101449275 |
| Stepglm[backward]+Enet[alpha=0.4] | 0.685099846390169 | 0.966386554621849 | 0.90755973364669 |
| Stepglm[both]+Enet[alpha=0.3] | 0.685099846390169 | 0.957983193277311 | 0.90638464551508 |
| Stepglm[backward]+Enet[alpha=0.3] | 0.686635944700461 | 0.966386554621849 | 0.906972189580885 |
| Stepglm[both]+glmBoost | 0.691244239631336 | 0.957983193277311 | 0.893654524089307 |
| Stepglm[backward]+glmBoost | 0.69431643625192 | 0.949579831932773 | 0.894242068155112 |
| Stepglm[both]+Enet[alpha=0.5] | 0.685099846390169 | 0.957983193277311 | 0.90677634155895 |
| Stepglm[backward]+Enet[alpha=0.5] | 0.689708141321045 | 0.974789915966387 | 0.90520955738347 |
| glmBoost+RF | 0.757296466973886 | 0.882352941176471 | 0.858793576184881 |
| RF | 0.763440860215054 | 0.865546218487395 | 0.866039952996475 |
| Stepglm[both]+SVM | 0.616743471582181 | 0.781512605042017 | 0.791421856639248 |
| Stepglm[backward]+SVM | 0.616743471582181 | 0.781512605042017 | 0.785056795926361 |
| Lasso+RF | 0.752688172043011 | 0.873949579831933 | 0.849784567175872 |
| Stepglm[both]+RF | 0.691244239631336 | 0.92436974789916 | 0.868194281237759 |
| LDA | 0.608294930875576 | 0.949579831932773 | 0.870740305522914 |
| glmBoost+LDA | 0.671274961597542 | 0.949579831932773 | 0.875244810027419 |
| RF+LDA | 0.698924731182796 | 0.915966386554622 | 0.877594986290638 |
| Stepglm[both]+LDA | 0.660522273425499 | 0.966386554621849 | 0.890325107716412 |
| Stepglm[backward]+LDA | 0.660522273425499 | 0.966386554621849 | 0.890325107716412 |
| Lasso+LDA | 0.649769585253456 | 0.941176470588235 | 0.875048962005484 |
| Stepglm[backward]+RF | 0.69431643625192 | 0.92436974789916 | 0.86623580101841 |
| XGBoost | 0.722734254992319 | 0.899159663865546 | 0.797688993341167 |
| Lasso+XGBoost | 0.577572964669739 | 0.73109243697479 | 0.794947121034078 |
| glmBoost+XGBoost | 0.715821812596006 | 0.928571428571428 | 0.807187622405014 |
| RF+XGBoost | 0.6321044546851 | 0.831932773109244 | 0.851253427340384 |
| Stepglm[both]+XGBoost | 0.668202764976959 | 0.983193277310924 | 0.894829612220917 |
| Stepglm[backward]+XGBoost | 0.715821812596006 | 0.928571428571428 | 0.807187622405014 |
| NaiveBayes | 0.751152073732719 | 0.890756302521008 | 0.800235017626322 |
| Lasso+NaiveBayes | 0.734254992319508 | 0.848739495798319 | 0.823736780258519 |
| glmBoost+NaiveBayes | 0.76036866359447 | 0.840336134453782 | 0.85996866431649 |
| RF+NaiveBayes | 0.758832565284178 | 0.815126050420168 | 0.884841363102233 |
| Stepglm[both]+NaiveBayes | 0.717357910906298 | 0.865546218487395 | 0.77692910301606 |
| Stepglm[backward]+NaiveBayes | 0.717357910906298 | 0.865546218487395 | 0.77692910301606 |

**Supplementary Figure 1**

| 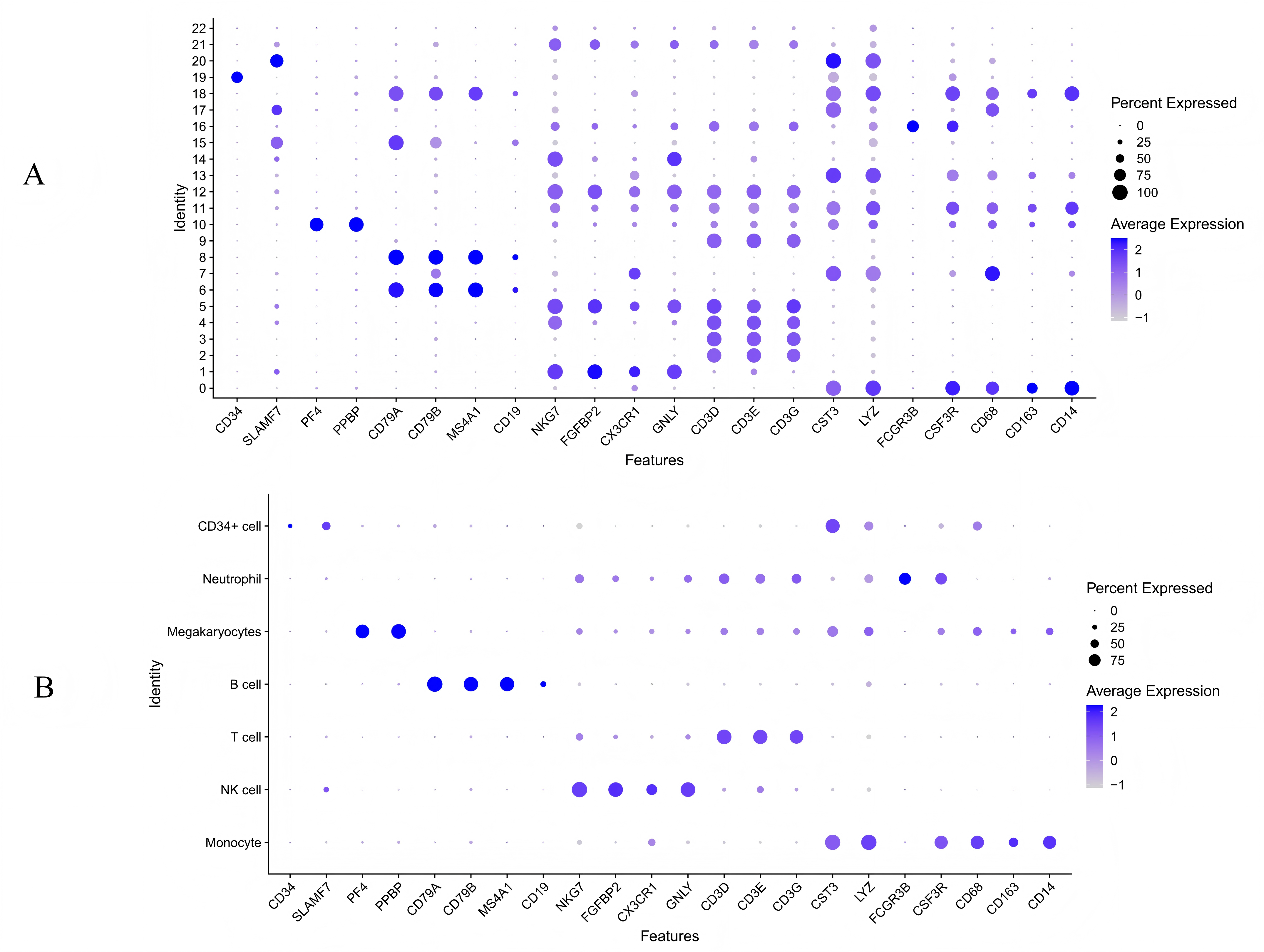 |
| --- |
| **Supplementary Figure 1.** Marker-gene expression used for cell-type annotation in the single-cell dataset GSE269269. |
